# Epidemiological Differences of COVID-19 Over the World

**DOI:** 10.7759/cureus.10316

**Published:** 2020-09-08

**Authors:** Abida Sayed, Krishna Teja Challa, Sateesh Arja

**Affiliations:** 1 Department of Medicine and Research, Avalon University School of Medicine, Willemstad, CUW; 2 Medicine/Clinical Skills, Avalon University School of Medicine, Willemstad, CUW

**Keywords:** coronavirus, covid-19, bcg vaccine, bacillus calmette guerin vaccine

## Abstract

Background

Coronavirus disease 2019 (COVID-19), originally, from Wuhan, China, has now spread to most countries across the globe and devastated global healthcare systems. The impact of this disease has, however, shown baffling variations in prevalence in different regions of the world. The aim of this short review is to identify differential national COVID-19 prevalence of COVID-19, as well as to suggest these epidemiological differences.

Methods

A review of studies was conducted using PubMed and Google Scholar search engines. Search tactics were centered on COVID-19 (“COVID-19” AND “coronavirus”) and BCG vaccination (“BCG vaccination” OR “Bacillus Calmette-Guérin” OR “vaccine”)

Results

It is found that national prevalence differences may be linked with BCG childhood immunization history. A statistically insignificant difference was observed in COVID-19 prevalence when comparing countries with a BGC policy and countries without it (P> 0.05). This inconclusivity suggests the influence of confounders in this study.

Conclusions

National differences in COVID-19 cases can be attributable to immunologic regulations, such as BCG vaccination protocols. Caution should be taken in establishing a correlation between COVID-19 prevalence and BCG vaccination, partly due to the weak quality of statistical data on COVID-19 related to poor testing rates in countries with BCG vaccination policy. Nonetheless, the analysis of the epidemiological aspects of COVID-19 will shed light on future efforts towards effective control and prevention.

## Introduction

A novel coronavirus referred to as coronavirus disease 2019 (COVID-19) or severe acute respiratory syndrome 2 (COVID-19 or SARS-COV2) continues to devastate the world. Currently, there are a total number of 26,795,708 confirmed COVID-19 cases recorded with 878,963 deaths worldwide as of 09-05-2020 [1]. The exponential growth in case numbers has shaken the healthcare and economic systems worldwide with alarming regional discrepancies being observed [2]. Eastern regions with relatively fragile healthcare systems show lower than expected incidence and mortality rates [3]. Such differences may possibly be due to cultural standards, palliation measures, and healthcare infrastructure. A recent study proposed that Bacillus Calmette-Guérin (BCG) vaccination provides some protection against viral disease. This vaccine has been utilized since the early 1920s; it is a safe vaccine that showed safeguarding against a variety of pathogens and viruses [4]. The association between BCG vaccination and reduction in COVID-19 severity that is consistently being observed may currently be insufficient to demonstrate a causal link between BCG vaccine and protection against COVID-19 infection. However, ongoing research is currently trying to determine BCG vaccination roles in containing and inferring protection against COVID-19 mortality and morbidity and may have impacted the prevalence patterns seen in some countries. If this theory holds true, it can lead to beneficial connotations regarding worldwide vaccination regimens. Its possible transient protection and unspecified immune system enhancement can be particularly useful in vulnerable populations as well as help in buying time while waiting for the availability of COVID-19 specific vaccines and other treatments [5]. It is imperative to study the regional differences in prevalence and mortality patterns with possible exposure parameters such as BCG vaccination that can give us insight into COVID-19 preventative measures and better preparedness for future epidemics. 

## Materials and methods

This review study was conducted using standard review methods. The main study objective was to report the mean prevalence of COVID-19 infection across 20 countries. Furthermore, this study also performed a comparative analysis between countries with established current BCG vaccination policies and those without such a policy. This was done using a two-tailed t-test. Data were represented using mean values, standard deviation, and standard mean error. All statistical analysis and calculations were conducted using GraphPad (GraphPad Software Inc., California, USA). All Figures were generated using ChartGo software. COVID-19 related statistics were extracted from worldometers (https://www.worldometers.info/coronavirus/) according to the update on September 5, 2020 [6]. The vaccination status of each country was taken from the World Health Organization (WHO) BCG immunization report [7,8]. 

## Results

The analysis involved 20 countries. Characteristics included in the study are outlined in Tables 1-2. Table 3 represents the comparative analysis of COVID-19 prevalence in countries with BCG vaccination and countries without BCG vaccination as a policy. The statistical difference of COVID-19 prevalence in countries with BCG vaccination and countries without BCG vaccination as a policy was observed to have a mean difference of -189348.70 (95% CI: 414371.66 to 35674.26). There was a statistically insignificant difference in COVID-19 prevalence when comparing countries with a BGC policy and countries without it (P> 0.05; P=0.08).

**Table 1:** Countries that follow a national Bacillus Calmette-Guérin (BCG) vaccination policy and number of COVID-19 cases recorded

Country	Population	Total cases	Total deaths	Case fatality rate
1.India	1369 Million	4,020,239	69,635	1.7%
2.Pakistan	220.8 Million	297,512	6,335	2.1%
3.Brazil	210 Million	4,091,801	125,584	3.2%
4.Japan	126.9 Million	70,268	1,330	1.9%
5.Ireland	5 Millions	29,303	1,777	6%
6.Ecuador	17.50Millions	117,175	6,674	5.7%
7.Thailand	67.79Milions	3,431	58	1.7%
8. Saudi	34.14Million	319,141	4,015	1.2%
9.Indonesia	257 Million	187,537	7,832	4.2%
10.Chile	17.4Milllion	418,469	11,494	2.7%

**Table 2:** Countries that did not have or ceased national Bacillus Calmette-Guérin (BCG) vaccination policy and their recorded cases

Country	Population	Total cases	Total deaths	Case fatality rate
1.USA	329.45 Million	6,389,057	192,111	3%
2.Italy	60.3 Million	274,644	35,518	13.1%
3.Spain	47.1 Million	517,133	29,418	6.1%
4.Germany	83.02 Million	250,281	9,401	3.8%
5.France	67.0 Million	309,156	30,724	10%
6.Austria	9.01 Million	28,729	735	3%
7.Belgium	11.23 Million	86,544	9,899	12%
8.UK	66.4 Million	342,351	41,537	13%
9.Switzerland	8.48 Million	43,532	2,013	5.3%
10.Norway	5.42 Million	11,231	264	2.8%

**Table 3:** Comparative analysis between COVID-19 prevalence in countries with BCG vaccination policy and countries without Bacillus Calmette-Guérin (BCG) vaccine policy (per 100,000)

	Mean of total current cases (Per 100,000)	Standard Deviation	Standard Error Mean
Countries with BCG Vaccine Policy (N=10)	9,55,488	1639640.22	1800885
Countries without BCG Policy (N=10)	8,25,266	1961802.67	3188913

## Discussion

The lower than expected COVID-19 cases in countries with poor health care systems is surprising. Statistical data on BCG vaccination policies by country may show a potential impact on the transmission of COVID-19 [9]. Our study demonstrated an insignificant relationship between COVID-19 prevalence and BCG regulations. The lowest prevalence was seen in Thailand, Indonesia, and Norway while the highest case numbers were seen in the United States (USA), Italy, and, Spain. Overall, in countries with proven BCG vaccine protocols in place, there was an insignificantly lower number of COVID-19 cases compared to those countries without such protocols (P > 0.05) The country hardest hit by this infection is the USA.

Many nations have adopted compulsory BCG vaccination protocols for prophylaxis of tuberculosis (TB) over the past few decades which at the time was a major threat [10]. The majority of countries subsequently agreed to continue the policy, at least not too long ago (China, Ireland, Finland, and France). Other regions decided to cease the policy when TB was no longer a threat (Australia Spain, Ecuador). There were some countries that did not warrant a policy at all (USA, Italy, Lebanon). Although Iran has a current BCG vaccination policy, it can be convinced that these vaccine strains produce short-lived immunity compared to other countries widely used older strains [11]. Consequently, there are adequate variations in BCG vaccination policy, either its presence or absence throughout various districts around the globe, making it feasible to derive such a correlation. 

The results obtained from this study are similar to a report by the World Health Organization stating “there is no evidence that the BCG vaccine protects people against infection with COVID-19 virus.” [12] WHO discourages vaccination as a means of prophylaxis against COVID-19 until further evidence is presented. Although there is evidence of the nonspecific immune protective effects, these benefits have not been fully characterized. Furthermore, a study by Hensel et al. [13] also showed a lack of significance when comparing COVID-19 incidence in countries with a BCG vaccine policy and countries without a current BCG vaccine regimen. They concluded that the observation of incident differences could be connected to population density, age, incidence of TB, regional population, COVID-19 testing rates interlinked with BCG protocols causing confounding effects. On the contrary, a study conducted by Gursel and Gursel [14] showed that case numbers in countries that have BCG vaccinate programs were significantly less than those without a vaccine program (P<0.0001). In addition, Hegarty et al. [15] and Mahase [16] considered a link between national BCG vaccine protocols and mortality rates. They concluded that nations with a population-wide BCG program appear to show a lower number of cases and deaths due to COVID-19 infection, possibly due to the confirmed immunological benefits of the vaccination. Shet et al. [17] also reported a pattern of COVID-19-related mortality amidst BCG-employed nations that was 5.8 times lower (95% CI 1.8-19.0) compared to countries without a vaccination program. The discrepancies in these studies further highlight the difficulty of obtaining valid statistical data and indicates a need for further research on this comparison. 

In general, a vaccine will provide its protection from distinct pathogens by its effector mechanism. A live attenuated vaccine such as BCG, an attenuated strain of Mycobacterium bovis, provides protection to pathogens causing respiratory infections [18,19]. There is enduring evidence that this vaccine induces unspecified mortality protection, the most commonly administered vaccine worldwide, enticing approximately 38%-45% reduction in fatality rates [20,21]. Developed to combat TB, its mortality benefits stem from a reduction in neonatal sepsis and respiratory infections. The unspecified immune advantages of BCG have been recognized as early as 1970 at the time when BCG showed to enhance immunity against pathogens such as listeria and influenza in mice models [22,23]. The underlying mechanism for this defense caused by the BCG vaccination is thought to be mediated through the activation of innate immune memory, or trained immunity [24]. In a randomized placebo controlled human study conducted by Arts et al. [25], it was evident that BCG vaccine induces genetic reprogramming of monocytes and shown protection against few experimental viral infections with an underlying key role of interleukin-1 beta (IL-1𝛃) as a trained immune response. It can be contemplated that countries which continue BCG immunization can help in reducing the spread of COVID-19 better as compared to the countries which do not have BCG vaccination policies, although this is only speculation. 

The comparative data from this study is acknowledged as being observational and predicated on single prevalence with the possibility of several confounding factors that can limit this study design. Since prevalence is dependent on testing capabilities in each region and may not represent the true degree of the epidemic regionally; a clear conclusion cannot be drawn due to such limited testing and reporting of cases [26]. A major challenge seen with the COVID-19 crisis has been obtaining clean data. The majority of available data related to COVID-19 has been of poor quality. This poor quality of comparable case data is greatly impacted by a nation’s testing of the virus [27]. Therefore, the accuracy of prevalence, incidence, and other statistical values is of concern, limiting the validity of epidemiologic studies such as this. Nonetheless, during a pandemic with a rapid and exponentially growing disease as COVID-19, epidemiologic studies can provide beneficial insight into public health policies and crisis management. 

## Conclusions

A clear variance in COVID-19 prevalence is seen between the Western and Eastern hemispheres. There is conjecture that BCG vaccines’ protective activity may be linked to lower numbers of cases in Asian and African countries. Although this study showed inconclusive results, the validity of this hypothesis can only be concluded by comparing the prevalence and deaths from BCG vaccinated and unvaccinated countries throughout the entire duration of this pandemic. Future studies on BCG vaccination may provide better understanding on vulnerable populations and prevention efforts. 
